# Roles of microRNAs in acute lung injury and acute respiratory distress syndrome: mechanisms and clinical potential

**DOI:** 10.3389/fimmu.2025.1570128

**Published:** 2025-09-25

**Authors:** Pengyu Wang, Dingyuan Lai, Litong Jin, Yi Xue

**Affiliations:** ^1^ Department of Emergency Medicine, Taizhou Central Hospital (Taizhou University Hospital), Taizhou, Zhejiang, China; ^2^ Department of Pediatrics, Jiaxing Hospital of Traditional Chinese Medicine, Jiaxing, China

**Keywords:** acute lung injury, ARDS, microRNA, mechanism, pathogenesis

## Abstract

Acute lung injury (ALI) is characterized by a systemic and excessive inflammatory response triggered by various direct or indirect pathogenic factors, resulting in increased permeability of the alveolar-capillary membrane and the accumulation of fluid in the alveolar and interstitial spaces. Clinical symptoms include reduced lung compliance, respiratory distress, and severe hypoxemia that is difficult to manage. Acute respiratory distress syndrome (ARDS) represents a more severe form of ALI. The incidence of ALI/ARDS among critically ill patients is approximately 10.4%, with a mortality rate as high as 45%. MicroRNA (miRNA) is a small, non-coding RNA molecule approximately 22 nucleotides in length, which plays diverse roles in cellular functions and exerts a significant regulatory influence on disease progression. Research related to miRNAs, particularly in the context of ALI/ARDS, has increased in recent years due to its crucial involvement in the disease process. This article elucidates the molecular mechanisms of miRNA and outlines the current research advancements in ALI/ARDS, offering novel insights into the pathogenesis and potential clinical applications of this condition.

## 1 Introduction

Acute lung injury (ALI) is characterized by damage to alveolar epithelial cells and capillary endothelial cells, leading to diffuse pulmonary interstitial and alveolar edema due to incomplete respiratory function, resulting in severe clinical syndrome. In severe cases, it can progress to acute respiratory distress syndrome (ARDS) (1). The direct risk factors for acute lung injury (ALI) consist of severe pulmonary infection, drowning, pulmonary contusion, and pulmonary embolism. Indirect risk factors encompass sepsis, massive blood transfusion, trauma, pancreatitis, fat embolism, and cardiopulmonary bypass (2). At present, the pathogenesis of ALI/ARDS has not been elucidated, and it is of great clinical significance to actively explore the specific mechanism of the occurrence and development of ALI/ARDS. MicroRNA (miRNA) is a kind of small, non-coding, single-stranded RNA molecule with a length of about 22 nucleotides (3). miRNA binds to the 3’ untranslated region (3’ UTR) of target mRNAs to regulate gene expression, either by blocking translation or promoting mRNA degradation. In humans, over 2000 mature miRNAs are encoded by the genome. They perform diverse functions, including inhibiting cell proliferation and apoptosis, inducing DNA damage, regulating autophagy, modulating immune system activity, and influencing cancer progression (4–6).

MicroRNAs (miRNAs) are a class of highly conserved single-stranded Rnas with a length of about 22 nucleotides, which are involved in the physiological and pathological functions of a variety of diseases, including tuberculosis (7), ALI/ARDS (8), pulmonary fibrosis (9), hepatitis (10), cardiovascular disease and cancer (10). miRNA mainly binds to the 3 ‘-untranslated region (3’ -UTR) of miRNA and controls multiple pathways and various cellular processes, such as inflammation-immune response and cell-cell interactions (11). Currently, it is understood that the overactivation and recruitment of inflammatory cells in the lungs lead to the production of numerous proinflammatory factors. The interaction of these factors with effector cells is considered the primary pathophysiological change in ALI/ARDS (12, 13). As a crucial regulator of inflammation, miRNAs are believed to have a significant impact on ALI/ARDS. Research has shown that abnormal miRNA expression is commonly seen in ALI/ARDS. For instance, a clinical trial revealed that miR-150 levels were lower in the serum of ARDS patients and had a negative correlation with the acute Physiology and Chronic Health assessment (APACHE) II score (14), an indicator to evaluate the condition and prognosis of ICU patients. The elevated expression of miR-122 is correlated with the severity and prognosis of patients with Acute Respiratory Distress Syndrome (ARDS). Combining miR-122 with the APACHE II score provides a valuable assessment for predicting the prognosis of ARDS patients (15). Given that miRNAs play an important role in ALI/ARDS, miRNAs may become diagnostic indicators and therapeutic targets for ALI/ARDS (16). This article examines the role of miRNAs in the pathogenesis and progression of acute lung injury (ALI) and acute respiratory distress syndrome (ARDS), aiming to offer novel insights for the understanding, clinical diagnosis, and treatment of these conditions.

## 2 Biological synthesis of microRNA

In 1993, Lee et al. discovered a 22-nt small non-coding RNA, lin-4, in nematodes through genetic analysis. This RNA molecule possesses unique characteristics: it is short in length, lacks protein-coding capabilities, and is transcribed into a precursor RNA with a hairpin structure. This precursor RNA is further processed into a 20-nucleotide RNA molecule through a specific mechanism (17). Further studies showed that this RNA was an antisense translation inhibitor of the mRNA of heterochronic developmental timing pathway proteins (LIN-14,LIN-28) in nematodes translational repressor) (18, 19). The 3’ UTR sequence of mRNA can be complemented by small temporal RNAs, inhibiting translation. These small RNAs are normally involved in encoding proteins that are repressed by the lin-4 gene product during the larval stage. Mutations in the Lin-4 gene result in a loss of inhibition, leading to abnormal development in mutants. In 2000, Reinhart (20) discovered another small RNA molecule with post-transcriptional regulatory function, known as let-7. Named small temporal RNAs (stRNAs) for their precise temporal regulation, these molecules have since been identified in various species including humans, mice, and plants (21). In 2001, three research groups from different countries identified nearly 100 genes in nematodes (Caenorhabditis elegans), fruit flies (Drosophila melanogaster), and humans. As a result, this type of small RNA was collectively named microRNA (miRNA), sparking a surge in research interest. With the growing number of miRNA family members, miRNA libraries have been established globally to catalog and name new miRNAs. To date, over 3,000 species of miRNA have been documented.

**Figure 1:**
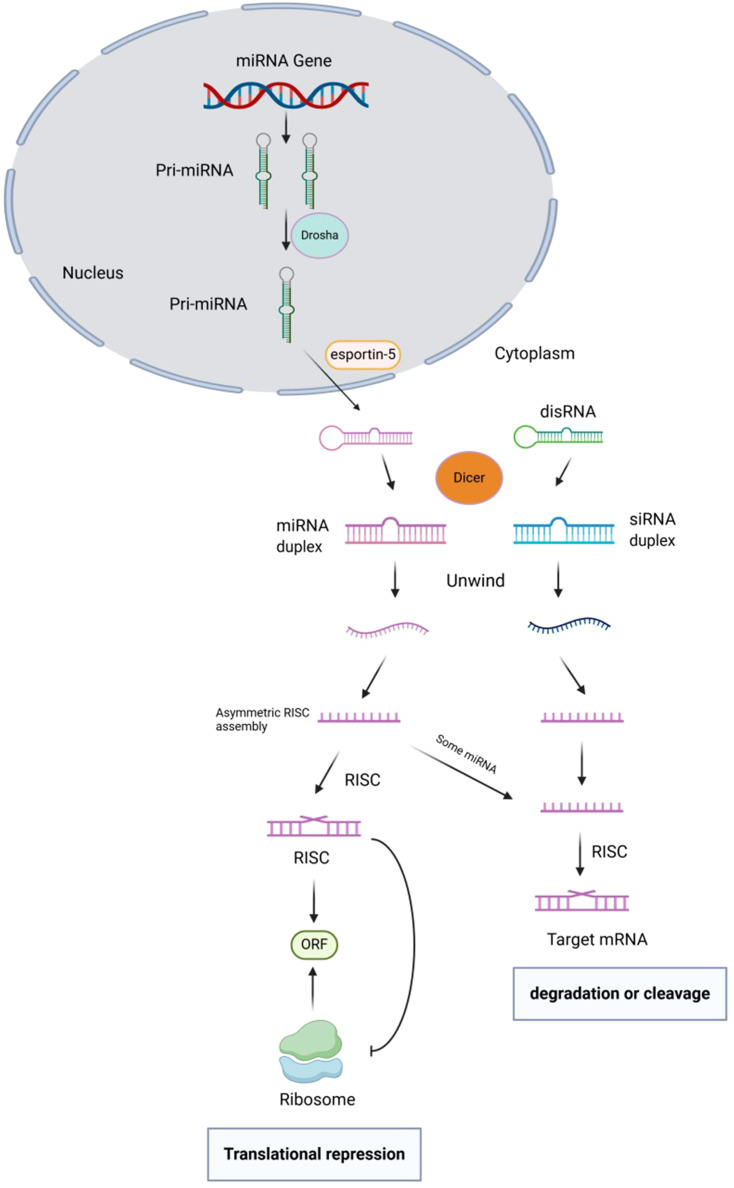
Biogenesis, function, and regulation of miRNAs.

Currently, there is a well-established understanding of the biosynthesis process of animal miRNA. Within the nucleus, the primary transcript of miRNA gene, known as pri-miRNA (ranging from 300–1000 bases in length), undergoes cleavage by the enzyme RNaseIII – Drosha. This cleavage results in the formation of a hairpin structure precursor miRNA (pre-miRNA) that is approximately 70–90 bases in length (22). After initial cleavage, the pre-miRNA is transported from the nucleus to the cytoplasm by the transporter exportin-5. It is then further cleaved by the Rnase III-Dicer enzyme to produce mature miRNA, which typically ranges from 20 to 24 nucleotides in length. The mature miRNA, along with other proteins in the RNA-induced silencing complex (RISC), leads to degradation or inhibition of translation of target mRNA. This process specifically targets precursor microRNAs (pre-microRNAs) with a hairpin structure, rather than a continuous double chain (23–25). The initial step in pri-miRNA cleavage involves the enzyme Drosha, resulting in the formation of a 70-nucleotide precursor hairpin structure (pre-miRNA). This structure contains the necessary sequences for processing on both sides of the hairpin. The double-stranded nature of pre-miRNA allows it to be recognized by the nuclear protein DGCR8, which interacts with Drosha to create the pri-miRNA processing complex (26). The Drosha enzyme contains an RNaseIII domain that cleaves the hairpin structure about 11 nucleotides from its base, releasing pre-miRNA. RIIIDa cleaves the 3’ strand of pri-miRNA, while RIIIDb cleaves the 5’ strand. CTT binding to RIIIDb is crucial for Drosha stabilization. This cleavage reaction not only produces the mature 3’ end of miRNA but also results in a 2 nucleotide protrusion at the 3’ end, a characteristic of RNase III cleavage. The cleavage occurs not at the base of the precursor hairpin but rather 11 nucleotides away from it. Pre-miRNAs need the Dicer enzyme in the cytoplasm for further processing, making their translocation out of the nucleus a critical initial step in maturation. The translocation of pre-miRNA from the nucleus to the cytoplasm is facilitated by a RanGTP/exportin-5 (Exp5)-dependent mechanism. Exp5 recognizes the 2 nucleotide protrusion at the 3’ end of pre-miRNA, facilitates its release from the Drosha complex, binds to it, and transports it to the cytoplasm for further processing. This process is regulated by the concentration of RanGTP, where high levels in the nucleus allow Exp5 to release pre-miRNA from the Drosha complex, while low levels in the cytoplasm prompt Exp5 to release pre-miRNA for binding with Dicer enzyme. The 3’ protruding end of the miRNA precursor aids in its transport. In animal cells, Drosha cleavage generates one end of the mature miRNA (3’ end), while Dicer cleavage at the other end (5’ end) occurs in the cytoplasmic envelope. Dicer, initially identified in studies on gene silencing by small interfering RNA (siRNA), also plays a crucial role in miRNA maturation (27–29). **Figure 1** shows the biogenesis, function, and regulation of miRNAs.

In fruit flies, nematodes, and mammals, Dicer plays a crucial role in the processing of long dsRNA into mature miRNAs. This process is akin to the production of double-stranded RNAs in RNA interference. Initially, exportin-5 transports pre-miRNA into the cytoplasm, where it is released due to the low concentration of RanGTP. Dicer then recognizes the double-stranded region of the pre-miRNA, which is phosphorylated at the 5’ end of the stem-loop base and exhibits a distinctive structure at the 3’ end. Dicer unwinds the two helices at the base of the stem-loop and cleaves both strands, resulting in the production of a 22nt-long, 5’-phosphorylated, siRNA-like, partially paired double-stranded RNA known as miRNA. This RNA duplex consists of mature miRNA and its complementary strand. Although Dicer can generate RNA double-stranded intermediates during pre-miRNA processing, these intermediates typically have a short lifespan and are challenging to detect (30).

## 3 Pathophysiological mechanisms of acute lung injury

The current conclusion suggests that alveolar cells play a crucial role in maintaining the stability of the alveolar structure. Disruption of the integrity of the alveolar capillary barrier or activation of the inflammatory response can result in alveolar dysfunction, leading to protein oedemas and the accumulation of inflammatory cells in the alveolar space. This cascade of events ultimately culminates in the development of ALI/ARDS (31, 32). During the acute phase seepage (0 to 7 days), respiratory dysfunction rapidly develops, manifesting in clinical symptoms such as shortness of breath, tachycardia, and respiratory alkalosis. Diffuse alveolar damage, caused by disruption of the epithelial-endothelial barrier, leads to excessive leakage of protein-rich fluid and blood cells into the interstitium and alveoli. Tissue damage triggers the migration of activated neutrophils, along with alveolar macrophages, platelets, and other inflammatory and fixed lung cells, contributing to inflammation (14, 33). Surfactant-producing alveolar type II cells (AECIis) are damaged by plasma proteins and proinflammatory factors, leading to surfactant loss in the alveoli. This loss causes atelectasis and decreased lung compliance. Dysfunction of epithelial ion channels reduces permeability needed to remove edematous fluid from the alveoli, worsening gas exchange. This leads to hyaline membrane formation, alveolar hemorrhage, and reduced lung compliance. Alveolar vascular injury also alters vasoconstrictor and vasodilator tone, leading to microthrombosis and pulmonary hypertension due to increased right ventricular afterload. Inappropriate mechanical ventilation and fluid overload can further worsen right ventricular dysfunction. Combined airway epithelial and endothelial injury causes perfusion mismatch and oxygenation difficulties (34, 35). However, the mechanisms of microvascular endothelial and alveolar epithelial injury are likely to be more complex and may vary depending on the intrapulmonary or extrapulmonary events that are triggered (36).

Within 5 to 7 days after injury, the exudative phase transitions smoothly to the proliferative phase. This phase is characterized by fibroblast and type II alveolar cell proliferation, as well as phenotypic changes and differentiation into type I alveolar cells. The regeneration of the epithelial layer helps clear edematous fluid in the lung, while remaining intraalveolar debris can be cleared by inflammatory cells, particularly macrophages. As the repair progresses, vasomotor tension decreases, leading to gradual improvements in oxygenation, pulmonary hypertension, and pulmonary compliance. In some patients, the proliferative phase may advance to the fibrotic phase, marked by diffuse fibrotic changes in lung tissue due to unsuccessful alveolar collagen clearance (14, 33, 37). Fibroproliferative changes may occur earlier than expected, with evidence suggesting collagen synthesis starting as early as 24 hours into the course of ARDS, potentially overlapping with inflammatory changes (36, 38).

## 4 miRNA: regulators in ALI/ARDS

Based on pathological and clinical manifestations, ARDS can be categorized into early and late lung injuries, representing the exudative phase and the fibroproliferative phase, respectively. The primary damage occurs in pulmonary vascular endothelial cells and alveolar epithelial cells (39). In the early stages, there is an increase in capillary permeability and the recruitment of leukocytes to the sites of inflammation. In the late stages of ARDS, there is the presence of fibrous and granulation tissue, alveolar type II cells, new blood vessels, and a disruption in the extracellular matrix during the healing process (38, 40). Recent studies have shown that the regulatory function of mirna in ARDS is mediated through a variety of physiological and pathological processes, such as inflammatory response and apoptosis (41). The role of lung epithelial cells and vascular endothelium in the pathogenesis of ARDS is well established. Various miRNAs play a role in epithelial cell apoptosis and endothelial dysfunction. In cardiopulmonary bypass-induced ALI, miR-320 was found to induce apoptosis and inhibit proliferation of A549 cells (human alveolar type II epithelial cells) (42). Li et al. identified Bcl-2 as the target gene of miR-181a, suggesting that downregulating miR-181a expression could decrease apoptosis in A549 cells treated with lipopolysaccharide (LPS) (43). Additionally, in a mouse model, miR-1246 was found to mediate LPS-induced lung endothelial cell apoptosis through partial inhibition of angiotensin-converting enzyme 2 (ACE2) (44). Shah.et al demonstrates that miR-34a regulates mechanisms-1 and the expression of p53 to control endothelial cell apoptosis and dysfunction (45). Furthermore, the delivery of miR-126 through exosomes originating from endothelial progenitor cells enhances the expression of tight junction proteins, thereby preserving the integrity of the alveolar epithelial barrier (31).

Lung inflammation is a significant contributor to acute lung injury (ALI). An imbalance between inflammatory and anti-inflammatory responses exacerbates the development of ALI/ARDS. In human lung epithelial cells, miR-454 was found to inhibit CXCL12 mRNA translation by targeting its 3’-UTR. The expression of miR-454 suppresses the production of inflammatory cytokines and enhances the integrity of the alveolar epithelial barrier (46). LPS-induced acute lung injury (ALI) was identified by elevated levels of the proinflammatory cytokines IL-6 and miR-181b. Previous studies have shown that miRNA-181b regulates the expression of p65 protein via a specific signaling pathway, thus playing a crucial role in the development of ALI (47). miR-140-5p has been shown to increase the expression of toll-like receptor 4 (TLR4) and MyD88 protein in human lung A549 cells, along with the activation of nf-kappa B. Up-regulation of miR-140-5p has been found to inhibit the inflammatory response in an acute lung injury (ALI) model by reducing the activation of the TLR4/MyD88/NF-κB signaling pathway (48). In ALI/ARDS induced by viral and bacterial lung infection, there was a significant increase in the expression of miRNA-200c-3p, along with a decrease in the expression of ACE2 protein. The overexpression of miR-200c-3p, dependent on Nf-κb, led to a reduction in ACE2 levels, subsequently increasing angiotensin II levels and resulting in lung injury (49). Some miRNAs (e.g., miR-140-5p, miR-200c-3p) not only suppress NF-κB signaling but are also transcriptionally induced by NF-κB, creating dynamic feedback loops that amplify or resolve inflammation. Also, miRNA-mediated signaling pathways exerted their roles by integrating mitochondrial cross-talk and regulating production of inflammasomes. miRNA-466 family molecules are secreted by the airway epithelium and regulate inflammatory responses through the NLRP3 inflammasome pathway (50). The inactivation of the Nlrp3 inflammatory corpuscle, mediated by vesicle delivery of micrornas, particularly miR-223 and miR-142, inhibits macrophage activation and lung inflammation (8). Yan et al. discovered that miR-155 is expressed in bone marrow-derived lymphocytes and is freely expressed in lymphatic lung parenchymal cells. They found that miR-155–2 promotes acute lung injury (ALI) induced by LPS through the Tie-2 channel in lung parenchymal lymphocytes (51). MiR-223 plays a crucial role in acute lung injury (ALI) induced by damage-associated molecular patterns (DAMPs). Specifically, miR-223 downregulates NLRP3 expression and inhibits IL-1β release, leading to a decrease in Ly6G+ neutrophils. This ultimately mitigates ALI caused by mitochondrial DAMPs (52). Studies have also indicated that miRNAs regulate inflammation through various pathways. For instance, research conducted by Yin et al. demonstrated that miR-34/449 suppresses inflammation by targeting IGFBP-3, consequently inhibiting autophagy (53).

Macrophages in the occurrence and development of inflammation and has an irreplaceable role in the fading. Of interest is the involvement of macrophages in initiating the lung repair process (54). The current study has shown that most experiments involving ALI and miRNAs have focused on macrophages. One study examined the processing of inactivated RAW264.7 cells by *Staphylococcus aureus* and found that the expression of miR-128 was associated with MyD88 UTR. MyD88 is a key downstream molecule in the Toll-like receptor (TLR) and interleukin (IL) 1 receptor signaling pathways. The interaction between miR-128 and MyD88 led to a significant downregulation of miR-128 expression and inhibition of the NF-κB signaling pathway (55). In RAW264.7 mouse macrophages, the binding of miR-92a to the 3’-UTR of phosphatase and tensin homologue (PTEN) resulted in decreased expression of miR-92a. Inhibition of miR-92a led to reduced inflammation in LPS-induced ALI mice by blocking the PTEN/AKT/nf-κB signaling pathway (56). Macrophages exhibit two phenotypes in response to environmental stimuli, one classically activated (M1) and the other alternative activated (M2) (57). IFN-γ and LPS stimulation led to the development of an M1-type phenotype characterized by elevated expression of iNOS and other genes linked to pathogen elimination, along with genes that regulate the inflammatory response to intracellular pathogens. Conversely, IL-4 and IL-13 triggered increased expression of arginase-1 (ARG-1) in M2 macrophages. These macrophages exhibited upregulation of genes associated with wound healing, clearance of dead/dying cells or tissues, and dampening of inflammation (58–60). M1 and M2 macrophages in ALI/ARDS pathophysiologic process plays a vital role (61). Lps-treated lung macrophages and U937 cell lines exhibited high expression of miRNA-34a, which was due to increased iNOS secretion by lung macrophages through the STAT3 pathway (62). Partial inhibition of Notch 1 miR-146a leads to the inhibition of macrophage M1 polarization and promotes the transition to the M2 phenotype. Furthermore, PPARγ, which is also targeted by Mir-146a for macrophage polarization, plays a role in driving the transition toward the M2 phenotype (63).

Fibrosis is a prominent characteristic of advanced Acute Lung Injury (ALI). Previous studies have demonstrated that miR-204 can reduce fibrosis and inflammation in the pulmonary alveoli by silencing IRF2 (64). Non-physiological stretching of alveolar type II (ATII) cells can induce fibrosis and expedite the epithelial-mesenchymal transition process, which is associated with miRNAs like miR-15b, miR-25, and let-7d (65). The down-regulation of miR-425 leads to the activation of the TGF-β signaling pathway by up-regulating lysine demethylase 6A (KDM6A), which in turn promotes lung fibroblast proliferation and ultimately results in fibrosis (66). Stem cell therapy has emerged as a popular non-invasive treatment for Acute Lung Injury (ALI), showing significant efficacy in animal models. Previous experiments have elucidated the biological process of miRNA regulation of Mesenchymal Stem Cells (MSCs). For instance, miR-132-3p has been shown to regulate the expression of ADAMTS-5, promoting the differentiation of rat MSCs into cartilage (67). Moreover, miR-302a and miR-34a have been identified as regulators of MSC proliferation. These miRNAs, along with Parp1, function as epigenetic switches that help maintain pluripotency. Interestingly, miRNA-302a and miRNA-34a have been found to have contrasting effects on PARP1 expression (68). Additionally, it has been confirmed that Wnt5a is a target gene of miR-374 in rat bone marrow mesenchymal stem cells. The Wnt5a/β-catenin signaling pathway plays a crucial role in regulating the proliferation and migration of transformed MSCs (69).

## 5 Mechanism of action of miRNAs in ALI/ARDS

ALI/ARDS primarily damages alveolar epithelial cells, vascular endothelial cells, and macrophages, resulting in alterations in cell morphology, structure, and function. As such, it is imperative to investigate the associated mechanisms at the cellular and molecular levels.

### 5.1 miRNAs associated with alveolar epithelial cells

Alveolar epithelial cells are crucial elements of the alveolar vascular barrier. Damage to these cells can result in a decline in barrier function, loss of epithelial integrity, decreased surfactant synthesis, impaired lung water clearance, and ultimately, pulmonary fibrosis. MicroRNAs play a role in regulating cell proliferation, apoptosis, and the expression of inflammatory factors by modulating various signaling pathways, thereby influencing the function of alveolar epithelial cells.

#### 5.1.1 Protective miRNAs

miR-145-5P was significantly down-regulated in lipopolysaccharide (LPS)-induced alveolar type II epithelial cells (ATII) (70). Additionally, miR-16 (71), miR-140 (72), and miR-140-5p were also significantly decreased in adenocarcinomic human alveolar epithelial cell (A549) (48). These miRNAs interact with the 3’ UTR of toll-like receptor 4 (TLR4) mRNA. TLR4, a pattern recognition receptor of LPS, is located in the cell membrane and cytoplasm (73). Upon LPS binding, it initiates a signaling cascade that activates the downstream NF-KB pathway (74). Therefore, the overexpression of miR-145-5p, miR-16, miR-140, and miR-140-5p can inhibit TLR4 expression, block NF-KB pathway activation, and reduce inflammatory factor expression, thereby alleviating acute lung injury (ALI). It was observed that miR-16 expression significantly decreased in hyperoxia-induced type 2 alveolar epithelial cells (AECII, T2AEC) (75), while miR-216a expression was reduced in LPS-induced A549 cells (76). Overexpression of miR-16 promotes cell proliferation and inhibits apoptosis, potentially through the TGF-β/Smad2 and JAK/STAT3 pathways (75). MiR-216a targets JAK kinase 2 (JAK2) to modulate JAK2/STAT3 and NF-KB signaling, inhibiting apoptosis, autophagy, and inflammatory cytokine release (76). Studies have shown that the TGF-β/Smad pathway primarily regulates cell growth, proliferation, differentiation, apoptosis, and migration (77), while the JAK/STAT pathway controls cytokine transcription, adhesion molecules, and inflammatory factors (78). Therefore, miRNAs can mitigate ALI/ARDS by influencing multiple signaling pathways (TGF-β/Smad, JAK/STAT, and NF-KB).

#### 5.1.2 Injurious miRNAs

miR-34a expression was significantly increased in LPS-induced ATII cells (79), T2AEC and murine lung epithelial-12 (MLE12) exposed to hyperoxia. The results confirmed that miR-34a could bind to forkhead box 03 (Fox03) (79) and 3’-UTR of angiopoietin-1 (Ang1) (80), which are related to autophagy. Previous studies have shown that FoxO3 can inhibit the activity of NF-kB (81), while Fang et al. (82) found that Ang1 can inhibit the activity of NF-kB in human T2AEC to restore the permeability of epithelial cells to proteins. So the miR - 34 a mediated by target control FoxO3 or Ang1 NF - KB pathways to regulate alveolar epithelial cell function. In the LPS-induced AEC model, Targeting B cell lymphoma 2 related A1 A1,BCL2A1) gene miR-326 activated the NF-KB signaling axis (83), and another study found that miR-300 targeted the inhibitor kappaB a (IkBa) protein and activated the NF-KB pathway in A549 cells treated with LPS (84). And in the resting state, NF - KB dimers with IkB protein in cytoplasm (85), after lung injury, cells by exogenous stimuli, NF - KB activation into the nucleus launched of the inflammatory response (86). The results showed that overexpression of miR-326 and miR-300 could increase the expression of pro-inflammatory factors and promote the apoptosis of alveolar epithelial cells by mediating the NF-KB signaling pathway, while inhibition of miR-326 and miR-300 expression could alleviate acute lung injury.

### 5.2 miRNAs associated with vascular endothelial cells

ALI induces depolymerization of cytoskeletal proteins in vascular endothelial cells, resulting in the loosening of intercellular connections. This leads to increased vascular wall permeability and the accumulation of inflammatory cells such as monocytes, lymphocytes, and multinucleated cells. MiRNAs target specific mRNAs in vascular endothelial cells, influencing endothelial cell homeostasis by modulating processes such as the cell cycle, apoptosis, cell layer permeability, and inflammatory signaling. This regulation ultimately impacts the function of lung injury associated with endothelial cells.

#### 5.2.1 Protective miRNAs

The expression of miR-339-3p, miR-539-5p and miR-33 was down-regulated in LMECs induced by LPS. miR-339-3p was confirmed to target annexin A3(Anxa3), inhibit AKT/mTOR pathway (87), while Anxa3 is involved in the regulation of cytoskeletal protein interaction, cell differentiation, proliferation, apoptosis and inflammatory response (88), and AKT/mTOR pathway also regulates cell apoptosis and inflammatory response (89).Luciferase reporter gene confirmed that Rho-associated coiled-coil conta-ining protein kinase 1, ROCK1) is a target gene of miR-539-5p (90), and ROCK1 is associated with oxidative stress and apoptosis in ALI (91). Therefore miR - 339–3 p and miR - 539–5 p reduce cell apoptosis of acute lung injury and the expression level of inflammatory factors. miR-33 is negatively correlated with receptor interacting protein 140(RIP140) (92), which is a co-activator of NF-KB. Overexpression of miR-33 reduced the inflammatory response of ALI by recruiting cAMP response element binding protein (CREB) to regulate the production of proinflammatory factors (93).

#### 5.2.2 Injurious miRNAs

After LPS and hyperoxia stimulated endothelial cells (EC), it was found that Mir-34a-5p-mediated endothelial dysfunction was related to the decreased expression of histone deacetylase 1(SIRT1) and increased expression of p53 (45), SIRT1 promotes the entry of apoptotic protein Bax into mitochondria by mediating p53, leading to mitochondrial oxidative stress damage (94, 95). LPS significantly stimulated the expression of miR-1246 and miR-92a in pulmonary microvascular endothelial cells. Because miR-1246 targets the angiotensin-converting enzyme 2(ACE2) gene (44), ACE2 is through pyrolysis Ang II produces angiotensin 1–7 to Ang II inactivation (96), And Ang II receptors and regulating oxidative stress, inflammatory reaction and apoptosis to relieve acute lung injury (97). The results showed that inhibition of miR-34a-5p or miR-1246 expression could inhibit oxidative stress, alleviate endothelial cell apoptosis, and reduce the expression level of inflammatory factors. Inhibition of miR-92a increased the expression of its target gene integrin a5(ITGA5) (98), ITGA5 plays a key role in cell adhesion, proliferation, and migration (99). The results also confirmed that ITGA5 significantly increased pulmonary microvascular endothelial cell migration, enhanced angiogenesis, improved endothelial cell function, and reduced the release of pro-inflammatory cytokines. Studies have shown that the above effects of miR-92a and ITGA5 may be related to PI3K/AKT and NF-KB pathways (100).

### 5.3 miRNAs associated with macrophages

Macrophages play diverse roles including phagocytosis and secretion. During the initial phase of acute lung injury, macrophages release various cytokines to trigger the inflammatory response. Numerous studies have demonstrated the involvement of miRNAs in immune regulation, monocyte development, differentiation, proliferation, and other key processes.

#### 5.3.1 Protective miRNAs

The expression levels of miR-497 and miR-30b-5p were found to be reduced in mouse peritoneal macrophage cells (RAW264.7) when induced by LPS. Inhibition of miR-497 expression led to an increase in interleukin 1 receptor associated kinase 2 (IRAK2) expression, while simultaneously inhibiting the expression of proteins in the NF-kB pathway (101). In the early stages of TLR, both IRAK1 and IRAK2 play important roles, but in the later stages IRAK2 plays a key role (102). Previous studies have found that IRAK2 is involved in IL-1induced activation of the NF-KB pathway (103). miR-30b-5p is associated with the expression of suppressor of cytokine signaling 3, which is associated with the expression of suppressor of cytokine signaling 3. SOCS3) of 3 ‘UTR with (104), and a variety of cytokines mediated SOCS family (105), and negative regulation of SOCS3 JAK/STAT3 pathway mediated pulmonary macrophage inflammatory (106). Both miR-497 and miR-30b-5p have been shown to suppress the expression of inflammatory factors in acute lung injury (ALI). Lipopolysaccharide (LPS) exposure has been linked to decreased expression of miR-495 and miR-802 in alveolar macrophages. Research indicates that methylation of the miR-495 gene promoter leads to miR-495 degradation and activation of NLRP3, while preventing miR-495 degradation can mitigate the inflammatory response and pyroptosis in alveolar macrophages, ultimately alleviating ALI (107). You et al. (108) confirmed that miR-802 improved lung injury by targeting pellino E3 ubiquitin protein ligase family member 2(Peli2). Peli2 mediates the activation of NLRP3 by promoting the ubiquitination of NLRP3 induced by LPS, which is closely related to the occurrence and development of pulmonary inflammatory diseases (109). In conclusion, both miR-495 and miR-802 can mediate inflammation and ameliorate ALI through the NLRP3 signaling axis.

#### 5.3.2 Injurious miRNAs

In LPS-induced RAW264.7 cells, there was a significant increase in the expression of miR-92a and miR-34b-5p. It was observed that miR-92a is linked to the phosphate and tension homology deleted gene on chromosome ten. The PTEN 3’-UTR interacts with and inhibits the PTEN/AKT/NF-KB signaling pathway, thereby suppressing the inflammatory response (56). PTEN plays a key role in various processes such as proliferation, apoptosis and inflammation, and can inhibit the PI3K/AKT signaling pathway (110). Studies have confirmed that progranulin (PGRN) is the functional target of miR-34b-5p (111) and plays a key role in pathological processes such as inflammation and apoptosis (112, 113). miR-199a directly targets SIRT1 gene in alveolar macrophages (114), and SIRT1 has anti-inflammatory, anti-oxidation, inhibition of DNA damage and reduction of apoptosis in various types of cells (115, 116). Therefore, by inhibiting the miR - 92 - a and miR - 34 b - 5 p and miR - 199 - a expression, reduce the excessive inflammatory reaction and apoptosis on ALI, ALI survival rate in mice.

## 6 The roles of microRNAs in mitochondria damage associated ALI/ARDS

Mitochondria also play a pivotal role in ALI/ARDS by interacting with microRNA (miRNA). Based on current evidence, mitochondria are central hubs in ALI and ARDS, orchestrating metabolic dysfunction, inflammation, and cell death. Mitochondrial quality control systems, including biogenesis, dynamics, and mitophagy, interact with novel programmed cell death forms such as pyroptosis, ferroptosis, and cuproptosis, shaping tissue damage and repair processes. Mitochondrial impairment, characterized by disrupted energy metabolism (e.g., TCA cycle collapse), excessive reactive oxygen species (mtROS), and unbalanced fission/fusion dynamics, directly amplifies lung damage. miRNAs, often shuttled via extracellular vesicles (EVs), regulate these processes by targeting mitochondrial pathways: Mitochondrial DNA (mtDNA) released as a damage-associated molecular pattern (DAMP) via EVs or necroptosis activates TLR9, fueling inflammasomes (e.g., NLRP3) and endothelial injury. This mitochondria-miRNA crosstalk represents a promising target for precision interventions in ALI/ARDS. In this study, several studies have provided the experimental data of the involvement of mitochondria in miRNA functioning of ALI and ARDS.

Feng et al. (52) reported that miR-223 reduced the number of Ly6G+ neutrophils and suppressed the activity of the NLRP3 inflammasome to alleviate ALI induced by mitochondrial damage-associated molecular patterns (DAMPs). Mitochondria provide energy through oxidative phosphorylation, while p53 plays a central role in maintaining mitochondrial homeostasis (94). Mitochondria are involved in the regulation of apoptosis (programmed cell death), a process critical for precise cell number control and removal of unwanted/dangerous cells (95). Under stress conditions, p53 regulates mitochondrial repair, degradation, and apoptosis through multiple mechanisms. Dilip Shah et al. (45) showed that miR-34a prompted the translocation of p53 and Bax to the mitochondrial compartment by down-regulated miR-34a-targeted sirtuin-1 (SIRT-1), which disrupted the mitochondrial membrane potential, caused cytochrome C to be released into the cytosol, and thereby triggered a cascade of mitochondrial-mediated apoptosis in the lungs.

A recent study showed that a nanocarrier‐mediated synergistic miRNA‐127 antagonist exerted an anti‐inflammatory effect on ALI by restoring the mitochondrial functions of target cells (117). Almaz Zaki et al. (118) demonstrated that elevated miR-495 might represent a new target for future therapies in ALI by improving mitochondrial function. In line with these findings, Zhang et al. (119) found that increased miRNA-21 expression could prevent and treat ALI by elevating mitochondrial membrane potential and reducing the expression of mitochondrial fission proteins Drp1 and Fis1. The above studies collectively demonstrated that mitochondria serve as central hubs targeted by miRNAs to regulate key processes like metabolic dysfunction, inflammation, and cell death in the pathogenesis of ALI/ARDS.

## 7 miRNAs are potential biomarkers for the diagnosis and prognosis of ALI/ARDS

Recent research has focused on investigating the roles of miRNAs in various cellular processes such as proliferation, differentiation, body growth, development, and metabolism. Studies have shown that miRNAs play a crucial role in organ development and function (120). During lung development, the expression of miR-17–92 is initially high but decreases as development progresses. This decrease in expression can lead to increased proliferation and the maintenance of an undifferentiated phenotype in lung epithelial progenitor cells (121). miRNAs play a crucial role in the pathogenesis of lung cancer and the suppression of tumor cell growth. Madhu et al. (122) discovered that the let-7 microRNA family exhibits low expression levels in lung cancer, leading to a potential decrease in the disease burden. Boeri et al. (123) illustrated that the expression levels of miRNAs in lung tissue and plasma can serve as predictive markers for lung cancer development and metastasis, offering both theoretical and clinical implications for early detection of the disease.

The expression of specific miRNAs in serum is closely associated with various human diseases. Recent research has focused on using miRNAs as disease markers, with serum miRNAs offering advantages such as specificity, ease of detection, and high sensitivity. Combining multiple miRNAs to create an equation for accurate disease prediction is considered a valuable diagnostic approach. A significant number of patients with severe trauma or multiple traumas develop ALI/ARDS, emphasizing the importance of real-time evaluation and management. Current clinical and biochemical indicators used in intensive care units often fall short in assisting medical professionals. Studies have indicated significant changes in certain miRNAs in patients with ARDS, both in serum and cells, with varying degrees of miRNA expression alterations observed in different diseases. For instance, miR-21 is up-regulated in acute kidney injury due to renal ischemia/reperfusion, while miR-199a-3p shows lower expression levels in heart failure (124, 125). Another point is that miRNAs are conserved (126), which is reflected in the fact that miRNAs share some important sites with their precursor pre-miRNA, and the conservation may be involved in the recognition and cleavage of Dicer enzyme. The characteristics of these miRNAs indicate that we can use miR-NAs to evaluate and monitor patients with ALI/ARDS, and miRNAs can be used as biomarkers of the disease (127).

In a previous study, Yan et al. (128) investigated the impact of miRNAs on the prognosis of ARDS/ALI by conducting a clinical experiment involving 244 patients with ALI due to sepsis (44 severe sepsis, 102 sepsis) and 19 healthy volunteers. Patients with severe sepsis-induced ALI or ARDS exhibited notably higher APACHEII scores, 30-day mortality rates, and days of noninvasive ventilation compared to those with sepsis. The QRT-PCR results indicated that the levels of miR-155 and miR-146a in the plasma of patients with severe sepsis and sepsis-induced ALI were significantly elevated in comparison to the control group. Subsequent follow-up of 26 patients with increased miR-155 and miR-146a levels revealed that a majority of the patients experienced reductions in these markers, suggesting that their peak expression coincided with the most severe phase of ALI. ROC curves were utilized to assess the predictive value of miR-155 and miR-146a levels for 30-day mortality. The results demonstrated that the AUC for miR-155 and miR-146a in predicting 30-day mortality in sepsis patients were 0.782 and 0.733, respectively, slightly below the APACHEII score of 0.835 (P < 0.05).

Currently, clinical trials investigating the use of miRNAs as therapeutic targets primarily focus on tumors like lung cancer and liver cancer, with limited exploration in the context of ALI/ARDS diagnosis or treatment. There is a pressing need for clinical trials to evaluate the potential of miRNAs as therapeutic targets in ALI/ARDS. MiRNAs play a crucial role in the pathogenesis of ALI/ARDS and hold promising prospects for clinical intervention. Advancements in human genome data and delivery vectors may pave the way for the translation of miRNAs as therapeutic targets into clinical practice for ALI/ARDS. The targets and function of microRNAs in ALI/ARDS were summarized in **Table 1**.

**Table 1:** Targets and function of microRNAs in ALI/ARDS.

Type	Micro RNA	Target	Function	Signaling pathway	Reference
Protective	miR-145-5p	TLR4	anti-inflammatory	NF-κB	(70)
	miR-16	TLR4	anti-inflammatory	NF-κB	(71)
	miR-140	TLR4	anti-inflammatory	NF-κB	(72)
	miR-140-5p	TLR4	anti-inflammatory	NF-κB	(48)
	miR-16	TGF-β	anti-inflammatory	JAK/STAT3	(75)
	miR-216a	JAK2	anti-inflammatory	JAK/STAT3,NF-κB	(76)
	miR-339-3p	Anxa3	anti-inflammatory	AKT/mTOR	(87)
	miR-33	RIP140	anti-inflammatory	NF-κB	(92)
	miR-497	IRAK2	anti-inflammatory	NF-κB	(101)
	miR-30b-5p	SOCS3	anti-inflammatory	JAK/STAT3	(104)
	miR-495	NLRP3	anti-inflammatory	/	(107)
	miR-802	NLRP3	anti-inflammatory	/	(108)
Adverse	miR-34a	Foxo3/Ang1	inflammatory	NF-κB	(79)
	miR-326	BCL2A1	inflammatory	NF-κB	(83)
	miR-300	IKBa	inflammatory	NF-κB	(84)
	miR-34a-5p	SIRT1	inflammatory	/	(45)
	miR-1246	ACE2	inflammatory	/	(44)
	miR-92a	ITGA5	inflammatory	PI3K/AKT,NF-κB	(56)

## 8 Other interacted proteins or pathways

**Figure 2:**
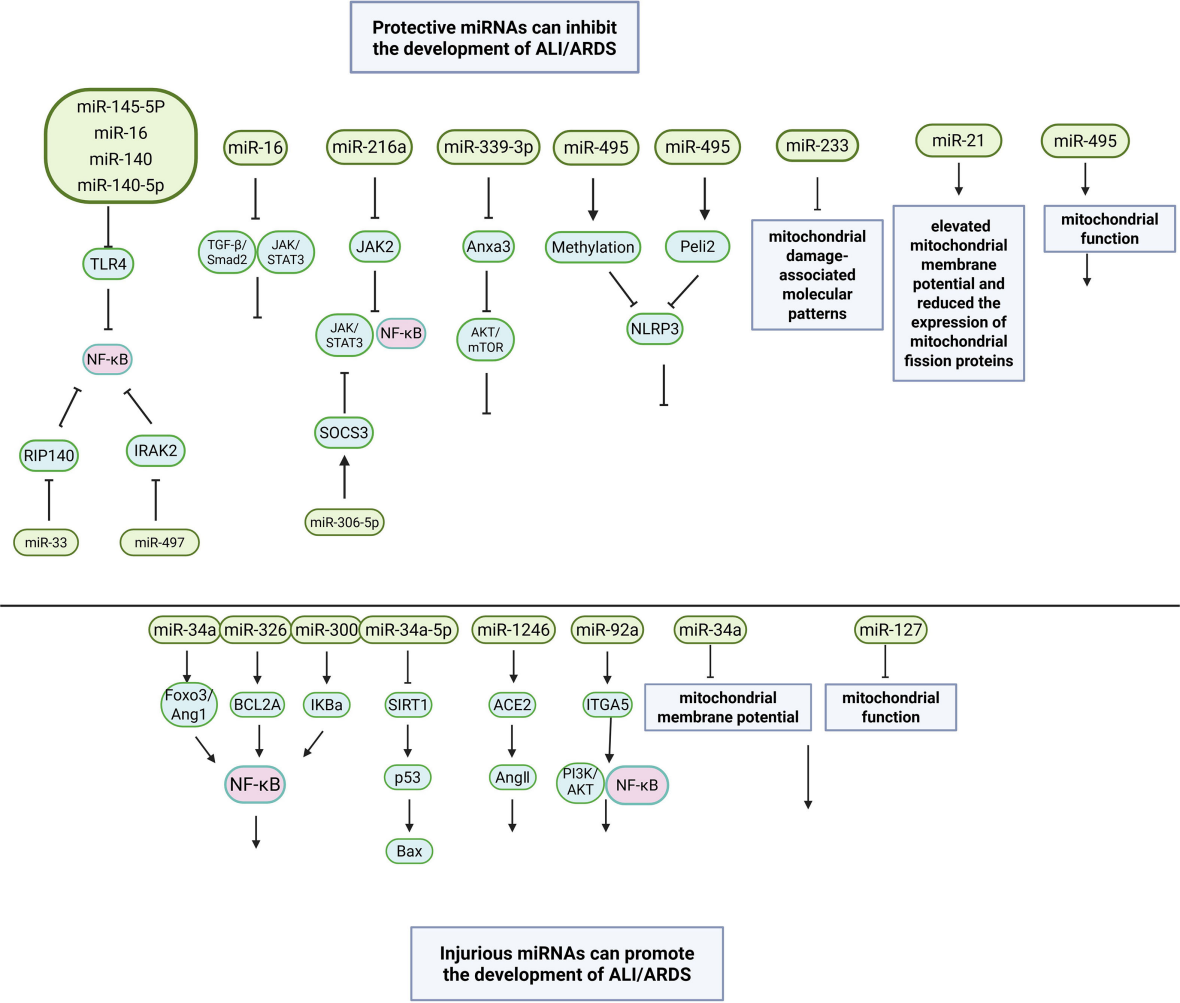
The molecular mechanisms of miRNAs in occurrence and development of ALI/ARDS.

LncRNAs also play important roles in ALI or ARDS (129). For example, lncRNA ZFAS1 regulates the inflammatory responses in sepsis-induced ALI via mediating miR-193a-3p (130). Extracellular vesicles (EVs), such as exosomes, can carry miRNAs and other bioactive molecules between cells (131). In ALI and ARDS, they can mediate cell-to-cell communication and affect the inflammatory process. Exosomes from macrophages can transfer miRNAs to alveolar epithelial cells, regulating their function (132). EVs and miRNAs affect the inflammatory process, including activation or inhibition of inflammatory signaling pathways, such as the NF-κB pathway (133). **Figure 2** displays the molecular mechanisms of miRNAs in occurrence and development of ALI/ARDS.

## 9 Summary and prospectives

The etiology of ALI/ARDS is complex. Despite significant advancements in understanding its pathogenesis, the exact mechanisms remain incompletely understood. Currently, uncontrolled inflammation is widely recognized as the primary pathophysiological alteration in ALI/ARDS (134–136). Inhibition of uncontrolled inflammation is a crucial strategy for treating ALI/ARDS. Evidence suggests that miRNAs play a significant role in regulating the inflammatory response in ALI/ARDS. Exploring miRNAs associated with the inflammatory signaling pathway of ALI/ARDS is clinically important for early diagnosis and treatment. This article delves into the role of miRNA, particularly miR-21, in the pathogenesis of ALI/ARDS and presents novel treatment approaches. While ongoing research continues to update our understanding of miRNA regulation in ALI/ARDS, current findings already underscore its significance in the disease’s onset and progression. Regulation of the relevant miRNAs occurs at the cellular, receptor, signaling pathway, and gene transcription levels (137). The intricate regulatory mechanism of miRNA within signaling pathways holds promise as a therapeutic agent or drug target for various pathological processes. Evidence suggests that changes in specific miRNAs levels are linked to different diseases, making miRNAs potential biomarkers for conditions like cancer and cardiovascular diseases. Notably, miRNAs are pivotal in regulating inflammatory responses, with altered expression in ALI/ARDS. Some miRNAs have emerged as novel biomarkers for diagnosing and predicting outcomes in ALI/ARDS. Multiple clinical cohorts have validated specific miRNA signatures for ALI/ARDS prognosis, including miR-122, miR-150, miR-155, and miR-146a. Challenges in delivery standardization, manufacturing scalability, and regulatory pathways are critically analyzed, providing a framework for clinical implementation. Consequently, miRNAs are being explored as potential therapeutic targets in diseases, with ongoing clinical trials testing miRNA drugs. This article elucidates molecular mechanisms of miRNAs in ALI/ARDS, outlines research advancements, and proposes future directions including miRNA-ibrutinib synergies, PROTAC-mediated protein degradation, and single-cell-resolved miRNA heterogeneity mapping via scSTAR/scPDA. Clinical translation challenges (e.g., inhalable nano-delivery, trial design) are addressed with scalable solutions.

This review explores the involvement of miRNAs in the development of ALI/ARDS. Numerous studies have shown that enhancing the levels of beneficial miRNAs and suppressing detrimental miRNAs can mitigate the symptoms of ALI/ARDS. Additionally, certain miRNAs have been identified as potential biomarkers for ALI/ARDS. Consequently, modulating the expression of miRNAs in ALI/ARDS could offer a promising avenue for future therapeutic interventions in lung injury.
